# ESTABLISHING A SHORT-TERM STUDY ABROAD COURSE ON AGING AND HEALTH CARE: FACILITATORS AND BARRIERS

**DOI:** 10.1093/geroni/igae098.2034

**Published:** 2024-12-31

**Authors:** Carla Hermann

**Affiliations:** Indiana University Southeast, New Albany, Indiana, United States

## Abstract

The population of older persons is growing in every country in the world. In the United States (US), the older population is increasing at an unprecedented rate and is becoming significantly more diverse. Population aging is one of the most significant social transformations of the twenty-first century. Every occupation will be impacted by this transformation; therefore, education of individuals across disciplines about aging is imperative. To address the demand for aging-related professionals, Indiana University Southeast developed and implemented an interdisciplinary short-term study abroad course on healthcare and gerontology for students of any major. Although this course was previously offered by other US universities in collaboration with the College of Health and Welfare at Jönköping University in Sweden, the course offered in May 2023 was the first time Indiana University Southeast was involved. There were multiple facilitators and barriers to establishing this short-term study abroad course. Facilitators included: collaboration with an established course and international experts; institutional support from the host university in Sweden; high interest in an international learning experience by the students; the course available to students of any discipline; offered over a short-term (2 week) period. Barriers to offering this course included: lack of institutional infra-structure to support study abroad; post-pandemic traveling concerns; lack of student knowledge regarding basic concepts of aging and healthcare. High impact educational practices such as study abroad can help meet the demand for professionals that are prepared to promote the well-being of the aging and diverse population.

